# *Point-of-Care* na Prática Clínica: Realidade Consolidada

**DOI:** 10.36660/abc.20230688

**Published:** 2024-02-16

**Authors:** Miguel José Francisco, Edgar Bezerra de Lira, Marcos Roberto Gomes de Queiroz

**Affiliations:** 1 Faculdade Israelita de Ciências da Saúde Albert Einstein Hospital Albert Einstein São Paulo SP Brasil Faculdade Israelita de Ciências da Saúde Albert Einstein (FICSAE) - Hospital Albert Einstein, São Paulo, SP – Brasil

**Keywords:** Ultrassonografia, Ecocardiografia, Medicina de Emergência, Faculdades de Medicina, Sistemas Automatizados de Assistência Junto ao Leito

A ultrassonografia e ecocardiografia com estudo Doppler representaram, ao longo das últimas décadas, avanços muito expressivos na prática médica em geral e, particularmente na medicina interna, promoveram verdadeira revolução no exercício diário sendo considerada uma ferramenta atualmente fundamental na prática clínica. Esta aplicação do método inclui tanto o cenário de atendimento de urgência quanto o ambulatorial.^1,2^

No cenário da urgência, a consagração inicial do protocolo FAST (Focused Assessment with Sonography in Trauma) para o atendimento do trauma de forma sistematizada possibilitou a popularização do uso do método e serviu de base para outras aplicações múltiplas. Numa escalada de ampliação de aplicação em medicina de urgência, a sequência inclui implantação dos protocolos RUSH (Rapid Ultrasound for Shock and Hypotension) e C.A.U.S.E. (Cardiac Arrest Ultrasound Exam),^3,4^ ganhando espaço progressivamente como ferramenta de múltiplas utilidades para uso no ensino médico, pesquisa e na prática médica ambulatorial.

A ultrassonografia atualmente tem papel importante no ensino nas disciplinas de Morfologia integrada à Imagem e Histologia, bem como à Fisiologia, destacando-se sua aplicação sistemática juntamente com metodologias ativas e dissecção eletrônica nos diversos sistemas do organismo. No currículo de alguns cursos médicos já se destaca como um diferencial importante, atuando como ponte segura entre o curso básico e os estágios clínicos, facilitando a autoconfiança, espírito de liderança e a capacidade de trabalhar com medicina baseada em evidência desde o início do aprendizado médico.^5^ No curso de graduação médica, a aplicação do método tem sido considerada progressivamente ao longo de toda a grade curricular. Em disciplinas do módulo básico e pré-clínico, representa uma ferramenta de integração entre os diversos ciclos do curso médico. É integrado com muita propriedade, por exemplo, na explicação prática do débito cardíaco, utilizando modelo vivo, na janela ecocardiográfica correspondente com o graduando identificando facilmente a via de saída do ventrículo esquerdo no estudo ecocardiográfico, modulando positivamente a integração morfofuncional. Desde a introdução do estetoscópio no início do século XIX por Laennec, a ultrassonografia representa o maior avanço da propedêutica médica.

Também há que se destacar que o futuro aponta que a ultrassonografia evoluirá a passos largos, com técnicas quantitativas para avaliação de fibrose e gordura hepática e, por outro lado, pela evolução dos equipamentos em miniatura de uso manual e alta precisão, facilitando a utilização desta tecnologia nas fronteiras do atendimento, cenário já amplamente consolidado e em expansão. Acrescente-se a isto a possibilidade de ser utilizado em exames à beira do leito e unidades avançadas, com baixo custo e ausência de radiação ionizante. Ademais, com a crescente aplicação de sistemas objetivos de pontuação dos achados, sistematizando o estudo em graus crescentes de complexidade, amplia-se de forma célere o uso do método ultrassonográfico.^6^

No atendimento hospitalar, seja no pronto atendimento, nas enfermarias ou nas diversas unidades de internação e terapia intensiva, destaca-se como ferramenta fundamental para garantir maior segurança nos procedimentos invasivos e na complementação do exame físico, determinando, por conseguinte, melhores resultados no contexto evolutivo.^7,8^

Particularmente no ambiente de atendimento ambulatorial, a ultrassonografia tem seu uso ampliado de forma definitiva. A aplicação desta tecnologia determina uma soma de sentidos pois condiciona a ampliação da propedêutica. O POCUS (point-of-care ultrasound) representa uma atividade diferente do exame ultrassonográfico completo que envolve documentação e relatório, sendo executado por especialista.^9^

Neste novo contexto, a ultrassonografia amplia sobremaneira as potencialidades da propedêutica em geral em todas as suas dimensões, particularmente na medicina interna, cardiologia e pneumologia, representando a transformação digital em que a tecnologia disponível incrementa muito as competências humanas, permitindo um avanço espetacular no diagnóstico morfológico unindo arte e ciência, potencializando o maior respeito ao paciente, que é o atendimento com utilização racional do recurso, materializando a incorporação do avanço tecnológico e propiciando melhor experiência ao paciente.^10,11^

Esta quebra de paradigma requer da comunidade médica brasileira e internacional algumas ações estruturantes, visando qualidade e segurança na sua aplicação:

1. Por se tratar de método operador dependente, há necessidade de elaboração de um currículo de treinamento e competências mínimas para sua correta aplicação pelos profissionais já egressos das escolas médicas;
2. Inclusão nos currículos médicos da ultrassonografia e ecocardiografia como método de ensino da anátomo-morfologia básica,^10^ face à realidade atual consequente às múltiplas experiências positivas de várias instituições de classe mundial;
3. Sistematização do ensino e aplicação da ultrassonografia no currículo médico, que já está aplicada na prática clínica e ocorre em diversas unidades de atendimento e de terapia intensiva;
4. Atuação das sociedades de especialidades, em conjunto com a Associação Médica Brasileira, para normatizar o uso desta ferramenta como avanço tecnológico definitivo.

Nos últimos anos houve uma explosão na literatura médica do número de artigos científicos sobre o uso do POCUS,^12^ sendo que o número de publicações relacionadas a este método cresceu de uma média de 8,8 publicações/ano no período de 2000 a 2004 para 134,8 publicações/ano de 2015 a 2019. Desta forma, fica claro que a incorporação da transformação digital na atividade da propedêutica já é uma realidade consolidada, cabendo à comunidade médica dar o devido olhar para este importante passo evolutivo no exercício da medicina baseada em precisão e evidência, com responsabilidade para unir a experiência à inovação,^13^ mantendo a somatória dos achados como um efeito potencializador para melhores resultados, possibilitando a realização de uma propedêutica mais completa para as próximas gerações.^14,15^
