# Supplementary material for: Point-of-Care na Prática Clínica: Realidade Consolidada
Source: Arq Bras Cardiol. 2024 Feb 16;121(1):e20230688. [Article in Portuguese] doi: 10.36660/abc.20230688 (PMC11081179; doi:10.36660/abc.20230688)
Supplement: Translation

# Translation: Point-of-Care in Clinical Practice: Consolidated Reality

**Authors:** Miguel José Francisco, Edgar Bezerra de Lira, Marcos Roberto Gomes de Queiroz

**Affiliations:** 1 São Paulo SP Brasil Faculdade Israelita de Ciências da Saúde Albert Einstein (FICSAE) - Hospital Albert Einstein, São Paulo, SP – Brasil

**Keywords:** Ultrasonography, Echocardiography, Emergency Medicine, Medical Schools, Point-of-Care Systems

Ultrasound and echocardiography with Doppler studies have represented, over the last few decades, highly noteworthy advances in medical practice in general. Particularly in internal medicine, they have promoted a true revolution in daily work and are currently considered a fundamental tool in clinical practice. This application of the method includes both urgent care and outpatient settings.^1,2^

In emergency scenarios, the initial establishment of the FAST (Focused Assessment with Sonography in Trauma) protocol for systematized trauma care made it possible to popularize the use of the method and served as a basis for multiple applications. In expanded application in emergency medicine, the sequence has included implementation of the RUSH (Rapid Ultrasound for Shock and Hypotension) and C.A.U.S.E. (Cardiac Arrest Ultrasound Exam) protocols,^3,4^ progressively gaining ground as a multipurpose tool for use in medical education, research, and outpatient medical practice.

Ultrasound currently plays an important role in teaching the subjects of Morphology integrated with Imaging and Histology, as well as Physiology, highlighting its systematic application together with active methodologies and electronic dissection in diverse systems of the body. In the curricula of some medical schools, it already stands out as an important differentiating factor, acting as a safe bridge between the basic course and clinical internships and facilitating self-confidence, leadership spirit, and the ability to work with evidence-based medicine from the start of medical education.^5^ In the undergraduate medical course, the application of the method has been considered progressively throughout the entire curricular framework. In subjects that are part of basic and pre-clinical modules, it represents an integrative tool between the different cycles of the medical course. It is integrated very appropriately, for example, in the practical explanation of cardiac output, using a live model in the corresponding echocardiographic window, with the student easily identifying the left ventricular outflow tract on the echocardiographic study, positively modulating morphofunctional integration. Since the introduction of the stethoscope at the beginning of the nineteenth century by Laennec, ultrasound has represented the greatest advance in medical propaedeutics.

It is also worth emphasizing that the future indicates that ultrasound will evolve in leaps and bounds, with quantitative techniques for evaluating fibrosis and liver fat, in addition to the evolution of miniature high-precision equipment for manual use, facilitating the use of this technology at the frontiers of care, a scenario that is already largely consolidated and in expansion. This is added to the possibility of use in bedside examinations and advanced units, with low costs and the absence of ionizing radiation. Furthermore, with the increasing application of objective systems for scoring findings, systematizing the study into increasing degrees of complexity, the use of the ultrasound method is rapidly expanding.^6^

In hospital care, whether in the emergency room, in wards, or in diverse hospitalization and intensive care units, it stands out as a fundamental tool to guarantee greater safety in invasive procedures and in complementing physical examination, thus leading to better results in the evolutionary context.^7,8^

Particularly in the outpatient care environment, the use of ultrasound has definitively expanded. The application of this technology determines multiple meanings, as it provides the conditions for expanding propaedeutics. POCUS (point-of-care ultrasound) represents a different activity from complete ultrasound examination that involves documentation and reporting and is performed by a specialist.^9^

In this new context, ultrasound greatly expands the potential of propaedeutics in general, in all dimensions, particularly in internal medicine, cardiology, and pulmonology, representing a digital transformation in which available technology greatly increases human skills, allowing a spectacular advance in morphological diagnosis uniting art and science, promoting greater respect for patients in the form of care with rational use of resources, incorporating technological advances, and providing better patient experience.^10,11^

This paradigm shift requires some structuring actions from the Brazilian and international medical community, aiming for quality and safety in its application:

1. Given that it is an operator-dependent method, there is a need to develop a training curriculum and minimum skills for correct application by professionals who have already graduated from medical school;
2. Inclusion in medical curricula of ultrasound and echocardiography as methods of teaching basic anatomy and morphology,^10^ considering the current reality resulting from the multiple positive experiences of several world-class institutions;
3. Systematization of teaching and application of ultrasound in the medical curriculum, which is already applied in clinical practice and occurs in diverse care and intensive care units;
4. Action by specialty societies, in conjunction with the Brazilian Medical Association, to standardize the use of this tool as a definitive technological advancement.

In recent years there has been an explosion in the number of scientific articles in the medical literature on the use of POCUS,^12^ with the number of publications related to this method growing from an average of 8.8 publications per year in the period from 2000 to 2004 to 134.8 publications per year from 2015 to 2019. Accordingly, it is clear that the incorporation of digital transformation in propaedeutic practice is already a consolidated reality, and it is up to the medical community to give due consideration to this important evolutionary step in the practice of precision and evidence-based medicine, with responsibility for uniting experience and innovation,^13^ maintaining the sum of findings as a potentializing effect for better results and making it possible to implement more complete propaedeutics for future generations.^14,15^
